# Supplementary material for: Are carbon emissions trading and green financial instruments synergistic? -Comprehensive quantitative research based on content analysis
Source: PLoS One. 2024 Mar 7;19(3):e0298601. doi: 10.1371/journal.pone.0298601 (PMC10919601; doi:10.1371/journal.pone.0298601)
Supplement: S1 Data — (ZIP) [file pone.0298601.s001.zip › Data/地级市Z_F工作报告.zip!/╜¡╦╒/╠⌐╓▌╩╨/└┤╫╘╣1⁄2╓┌║┼í╛┬φ┐╦╩2╛▌═°í┐_2012.docx]

各位代表：

现在，我代表市人民政府向大会作工作报告，请予审议，并请市政协委员和其他列席人员提出意见。

一、2011年工作回顾

过去的一年，在中共泰州市委的正确领导下，我们积极应对复杂多变的宏观形势，始终坚持科学发展，着力转变经济发展方式，全面加强社会建设，不断保障和改善民生，顺利完成市三届人大四次会议确定的目标任务，实现“十二五”经济社会发展良好开局。

经济平稳较快发展。预计全年实现地区生产总值2420亿元，可比增长12%左右，人均地区生产总值突破50000元。三次产业增加值分别为172亿元、1318亿元和930亿元，分别增长4.5%、12.7%和12.7%。财政总收入623.3亿元，一般预算收入218亿元，分别增长36.6%和27.6%。固定资产投资突破1000亿元，达1160亿元，增长22%。社会消费品零售总额644亿元，增长17%。农业生产稳定发展。实施高效农业“5218提升工程”，新增高效农业面积30万亩、省级现代农业产业园区4家，兴化市被表彰为全国粮食生产先进单位。加快农业产业化进程，楚龙面粉公司升级为国家级龙头企业，新增省级龙头企业12家，新建三大合作组织645家，农业利用“三资”45亿元。强化农产品质量建设，新增国家级农业标准化示范区2个，新获认证无公害农产品、绿色食品、有机食品481个。推进绿化造林工作，加强绿色通道建设和管护，完成造林面积7.7万亩。强化农田水利基本建设，靖江被表彰为全国先进市，泰兴、姜堰完成大型灌区改造。工业运行质态良好。预计规模以上工业完成销售5750亿元、利税680亿元、利润420亿元，分别增长30%、28%和30%。制定实施“新兴产业双倍增计划”、“传统优势产业升级计划”，预计新兴产业产值突破1000亿元，装备制造业实现产值2700亿元，增长28%，完成技改财务发生数1266亿元，增长26%，实施亿元以上重大技改项目210个，节能减排完成省定指标。民营经济加速发展，新增私营企业7430家、个体工商户25920个，净增私营个体经济注册资本318亿元。加强质量和品牌建设，新增中国驰名商标3件、国家级标准化良好行为企业10家，兴化市建成国家级不锈钢制品质量监督检验中心。建筑业发展步伐加快，预计完成总产值1540亿元，增长18%，我市被省政府命名为“建筑强市”，靖江成为又一个“建筑强县”。服务业发展力度加大。制定实施“服务业提速计划”，8大服务业集聚区、30强企业分别实现营业收入389亿元和200亿元，30个重点项目完成投资96亿元，服务业地税收入68.6亿元，增长32.5%，占地税总收入的56.7%。金融业持续发展，年末全市本外币存款余额2632.7亿元，贷款余额1804.5亿元，分别比年初增长11.8%和18.1%；兴业银行泰州分行开业，省信用再担保公司泰州分公司成立，新组建3家农村商业银行；发行企业债券28亿元、短期融资券和中期票据39亿元，泰凌医药、太平洋精锻成功上市。旅游业发展势头良好，预计全年接待游客1300万人次，实现总收入146亿元，分别增长20.3%和20.2%。

“双轮驱动”成效明显。有效组织“十大招商活动”，新落户世界500强企业4家，与央企签订合作项目12个。全年协议利用外资47.7亿美元，实际利用外资14.1亿美元。外贸进出口总额突破100亿美元，达105亿美元，增长22.3%，增幅居全省前列。创成国家船舶出口基地，新增13个“江苏省重点培育出口品牌”。外经工作取得新进展，实现营业额6.6亿美元，增长13%，正太集团、河海集团分别承建合同总额2.01亿美元的博茨瓦纳大学城项目和3.36亿美元的印尼围海造陆项目。继续举全市之力建设中国医药城，新落户企业超过200家，总投资2.3亿美元的阿斯利康、1.5亿美元的武田制药、10亿元的石药集团等一批重大项目落户开工，成功举办第二届中国（泰州）国际医药博览会。出口加工区二期加快建设，建成29万平方米厂房及配套设施，完成产品结构调整，即将正式投产。新能源产业园区获批国家火炬计划泰州新能源特色产业基地，成功举办太阳能产业高层论坛，新引进钒电池、晶硅电池片切割线等6个重大产业项目，协议总投资101亿元。沿江产业带建设纵深推进，泰州港吞吐量突破1亿吨，跨入全国大港行列，核心港区30万标箱集装箱码头基本建成，靖江市与中船重工投资40亿元共建船舶（游艇）科技产业园。里下河特色经济板块加快发展，南洋电缆、可可食品等项目落户姜堰、兴化。口岸工作实现新突破，泰兴港区沿江岸线全线开放。海关、国检、海事、边检为开放型经济发展作出新贡献，外事、侨务和港澳台事务工作取得新成绩。区域创新体系加快完善。我市连续4次被表彰为全国科技进步先进市，靖江、泰兴、高港被表彰为先进县（市、区）。预计全市高新技术产业产值突破2000亿元，增长30%以上。大力提升企业创新能力，新增省级创新型企业23家、市级以上高新技术企业60家、高新技术产品130个。加强政产学研合作，新增省级院士工作站3个、省级工程技术研究中心16家、外资研发机构14家、产学研联合体135个。强化科技服务，培育重大自主创新项目25项，入选国家科技重大专项8个、省成果转化资金项目9个。知识产权工作卓有成效，全国首家国家专利战略推进与服务中心落户运营，全省首家专利信息综合服务平台顺利开通，全市新获授权专利5036件，其中发明专利237件。深入实施“凤城千人计划”，引进创新创业人才团队64个、高层次人才1080名、海外优秀人才210名；医药高新区获批国家海外高层次人才创新创业基地，1人入选国家第六批“千人计划”，扬子江药业集团获批省第二批高层次人才创新创业基地。

城乡建设扎实推进。城市总体规划修编完成，上报国务院审批。加快实施十大工程，市区城建投入170亿元，中心城市品质和功能进一步提升。城区水生态环境封闭控制、城南污水处理厂改扩建等工程建成投运，长江大道、高港大道、永定东路改扩建等城市骨干道路竣工通车，大桥北接线、231省道海陵至高港段加快建设，医药高新区中央商务区、金融服务区等工程启动实施。万达广场建成运营后成为市区新的商业高地，茂业天地开工建设，希尔顿、碧桂园、泰州国际、世贸雅思等五星级酒店营业或即将营业。深化城市创建工作，国家卫生城市通过复核验收，全国双拥模范城市实现“三连冠”，国家环保模范城市复核通过省级预评估，节水型社会建设通过水利部中期评估，建成全省首个数字城市并被授予“全国数字城市建设示范市”，姜堰创成国家卫生城市。深入开展园林绿化工作，市区新增绿地面积85万平方米，绿地率36.7%，绿化覆盖率40.8%。继续打造“百姓城管”品牌，不断完善数字城管平台，城市管理水平进一步提升。深入推进新农村建设。认真办理农村新一轮实事，农村饮用水安全工程新增受益人口28万人，新建改造农村公路320公里、桥梁332座、户厕90374座。开展村庄环境综合整治，市区基本实现农村垃圾处理及卫生保洁全覆盖，靖江、高港通过农村河道疏浚省考核验收，全市秸秆能源化利用企业数量和秸秆利用量均居全省首位。实施全面小康“十百千提升工程”，创成达标村、示范村各100个。继续推进“125工程”，重点镇村建设完成投入120亿元。大力发展村级集体经济，预计新增集体经济年收入20万元以上村215个。推进市区村组（社区）股份制改革，基本完成街道社区区划调整工作，先后调整街道18个，撤村建居80个，调整社区99个。抓好全国供销合作总社改革发展联系点工作，新农村现代流通服务网络体系建设初见成效。出台实施“生态文明建设工程行动计划”，创成省生态乡镇34家，11个镇（街）国家生态乡镇创建工作通过环保部验收。加强耕地保护，有力保障重大项目建设用地，土地集约利用水平进一步提高。强化重大基础设施建设，长江大桥主桥全面贯通，机场工程主体竣工，宁启铁路复线电气化改造泰州段有序推进，卤汀河、泰东河拓浚工程进展顺利。

社会发展全面加快。加强文化泰州建设。科学发展观展示馆正式开馆；文化中心基本建成，规划展示馆开馆；五巷涵西、兴化金东门、姜堰北大街等历史文化街区建设取得阶段性成果，新增国家级、省级非遗项目2个和7个，天目山遗址被列为省首批八大遗址之一；海纪馆获批全国红色旅游经典景区，溱湖风景区跻身全省旅游景区“20强”，溱潼古镇创成4A级旅游景区；市文化创意产业园揭牌成立，施耐庵文学奖首次颁奖，成功举办水城水乡国际旅游节、梅兰芳艺术节等重大活动。推进教育现代化建设。学前教育加快发展，全市新增省级优质幼儿园15个；义务教育由基本均衡向优质均衡迈进，普通高中教育质量保持全省先进行列；职业教育、高等教育服务经济社会发展的能力进一步增强，泰兴创成省级职业教育创新发展实验区。全面实施基本药物制度，基层医疗机构综合改革进展顺利，市人民医院创成三级甲等医院，泰兴市人民医院创成三级乙等医院。全民健身运动深入开展，承办世界女子围棋锦标赛、全国木球锦标赛等一系列高水平赛事。全国新农村新家庭现场会在我市召开，市计生委被评为全国人口和计划生育系统先进集体。完成第六次全国人口普查，市统计局被评为“五五”统计普法全国先进单位。加强公民道德建设，放大“百姓”、“爱心”、“温心驿站”品牌效应，建成未成年人社会实践基地；深入开展创先争优活动，涌现出许年喜、周广智等全国先进典型；深化精神文明创建，黄桥镇、河横村、孝化村、董北村被评为全国文明村镇，扬子江药业集团、电信公司、供电公司、市级机关事务管理局、银监分局被评为全国文明单位。扎实推进社会管理创新。加强基层基础建设，形成三级联动的政法综治工作体系，“海陵模式”在全省推广；全面推行社会稳定风险评估制度，积极开展领导干部接访下访、信访积案化解和体制机制创新“三项活动”，努力从源头上预防和减少社会矛盾；深入推进平安泰州、法治泰州建设，严厉打击严重暴力刑事犯罪和多发性侵财犯罪，全市刑事案件总量有所下降，“清网行动”在册逃犯到案率居全省第一、全国地级市第一。“六五”普法有序开展，我市被表彰为全国“五五”普法先进单位，靖江被表彰为先进县（市、区）。开展“安全生产基层基础提升年”活动，安全事故持续保持“双下降”。理顺食品安全监管体系，加强药品安全监管，保障人民群众生命健康安全。强化应急保障，管理水平进一步提高。史志档案、社会科学、广播电视、新闻出版、气象、防震和民族宗教、妇女儿童、残疾人等工作继续加强，质监、工商管理为地方经济发展作出积极贡献，国防动员、民兵预备役和人民防空事业取得新成绩。

人民生活继续改善。预计城镇居民人均可支配收入23500元，农民人均纯收入11000元，分别增长16%和18%。市区最低工资标准上调至1140元，四市调整为930元。国家级创业型城市创建工作深入开展，在全国率先出台促进新生代农民创业就业20项政策，全年新增城镇就业6.38万人，城镇失业人员再就业3.31万人，新增创业人员5.71万人。社会保险扩面提质。新农合筹资标准大幅调至每人250元，全市筹集资金8.6亿元，参合率99.88%；学生儿童医疗保险覆盖市区在籍学生包括大学生，门诊大病和住院医保待遇提高到20万元；城镇居民医疗保险参保人数73万人，职工和居民医保报销比例平均提高到80%和70%；职工生育基本实现“零支付”，灵活就业人员工伤保险实现全覆盖。加强低保动态管理，城乡人均低保标准分别提高到每月380元和230元，靖江、高港低保标准实现城乡一体化。全面落实农村五保政策，全市集中供养率达65%。老龄事业加快发展，新增175家社区居家养老服务中心（站），为全市80岁以上老人发放“尊老金”1.25亿元，海陵区为70岁以上老人免费安装“电子保姆”。认真开展农副产品“保供抑价”工作，“平价菜篮子服务平台”正式运行，市区新建改造农贸市场5家，开工建设东风北路农副产品批发市场，建成平价商店48个。对困难群众发放物价补贴3900万元，有效减轻物价上涨对低收入群众生活的影响；建立孤儿基本生活保障自然增长机制，全市所有孤儿全部纳入保障体系；城乡居民医疗救助实现“两取消、两提高”。加快保障性住房建设，基本形成五级住房保障体系，新建各类保障性住房12977套（间）。住房公积金新增开户缴存职工5万人，当年归集额16.7亿元。市区公交事业加快发展，新辟、优化线路16条，新建、改造站台281座，新购车辆200辆，IC卡发行量突破10万张。

服务型政府建设深入开展。不断深化“三服务”活动和涉企非税收费专项治理，着力营造宽松的发展环境。坚持依法行政，深化政务公开，行政权力网上公开透明运行工作向乡镇延伸试点。优化行政审批服务，工程建设和餐饮服务领域实行“并联审批”。开通“12345”政府服务热线，认真办理市长信箱和群众来信，“行风热线”实现“声屏网报”联动运行，群众诉求得到有效解决。加快网上政府建设，大力开展网络问政，建成政府网站群，开办望海楼实名论坛。自觉接受市人大及其常委会的法律监督、工作监督和市政协的民主监督，认真办好人大代表建议和政协提案，满意（含基本满意）率100%，落实率56%。严格执行党风廉政建设责任制，推进惩治和预防腐败体系建设，强化行政监察和审计监督，加大纠风治乱和案件查处力度。加强公务人员职业道德培训，启动新一轮基层站所作风建设行动，开展市级机关效能建设大督查，行政服务水平得到新提升。

各位代表，过去一年成绩的取得，是全市人民在中共泰州市委的正确领导下，团结拼搏、开拓创新、克难奋进的结果，与市人大、市政协的监督支持密不可分。在此，我代表市人民政府，向全市广大干部群众，向全体人大代表、政协委员，向各民主党派、工商联、无党派人士、各人民团体和广大离退休老同志，向中央和省驻泰机构以及驻泰部队指战员、武警官兵和政法干警，向所有关心支持泰州发展的海内外朋友们，表示衷心的感谢!

在充分肯定成绩的同时，我们也清醒地看到，全市经济社会发展中还存在一些问题和不足。主要是，主要经济指标增幅回落，企业生产成本上升，经营压力加大；产业结构不尽合理，整体竞争力还不强，节能减排形势不容乐观；中心城市承载能力和服务功能需要进一步增强；物价涨幅有所放缓但仍处高位，推动城乡居民持续增收的难度加大；社会建设管理水平需要进一步提高，保障和改善民生工作与群众期待相比还有不小差距；少数部门存在的作风拖拉、办事不力、行政效能不高的问题还没有从根本上得到解决。这些问题，我们将在今后的工作中创新举措，努力加以解决。

二、2012年工作任务

各位代表，今年是党的十八大召开之年，也是我市经济社会发展至关重要的一年，做好全年政府工作具有十分重要的意义。

今年政府工作的总体要求是：深入贯彻科学发展观，全面落实市第四次党代会和市委四届二次全会决策部署，以争创全省转型升级示范区为抓手，坚持稳中求进、好中求快，大力推进开放创新，调整优化经济结构，统筹协调城乡发展，致力建设“三个名城”，加强和创新社会管理，着力提升公共服务，在“推进富民强市、建设美好泰州，向基本实现现代化迈进”的新征程上迈出坚实步伐。

全市国民经济和社会发展主要调控目标为：地区生产总值增长11%；财政总收入增长15%，一般预算收入增长15%；社会消费品零售总额增长16%；固定资产投资增长18%；实际利用外资12亿美元，外贸进出口总额增长8%；城镇居民人均可支配收入增长12%，农民人均纯收入增长12%；居民消费价格涨幅4%左右；城镇登记失业率控制在3.5%以内；全社会研发投入占地区生产总值比重1.9%，高新技术产业产值占规模以上工业比重34%；单位地区生产总值能耗下降率、主要污染物排放削减率达到省定目标。

今年将重点抓好六个方面的工作：

1. 坚持富民优先，增进民生幸福。以推进“城乡居民收入六年倍增计划”和健全“七大公共服务体系”为重点，把转方式、调结构、促转型和惠民生更好地结合起来，努力使经济结构与收入结构同步优化、产业结构与就业结构协调提升、经济增长与改善民生更趋一致。

大力推进就业创业。突出政策扶持的导向性、实效性，更大力度鼓励创业、扩大就业、促进增收。充分发挥就业在居民增收中的基础性作用，多渠道开发就业岗位，全面加强现代农民培育和新生代失业群体就业促进工作，进一步健全困难群众就业援助制度，加快形成“城乡一体、平台到村、联系到户”的就业服务体系。全年新增城镇就业6万人，创业带动就业15 万人。以创建国家级创业型城市为抓手，建立健全城乡创业政策、创业培训、创业服务的一体化体系，新增自主创业5万人。把农民收入倍增作为重中之重，千方百计拓宽农民增收渠道。转变企业发展方式，推动企业发展战略、发展动力、发展路径的加快转型，切实发挥企业在转型升级中的主体作用。深入实施“大企业（集团）培育”、“百企上市培育”计划，加快建设具有全球视野、战略思维、开放创新理念的企业家队伍，着力提升企业发展规模和层次，力争年销售收入50亿元以上企业达13家，新增上市企业2-3家；大力发展民营经济，突出扶持中小企业，扎实推进“六大工程”，促进企业健康发展、加快成长，全年新增注册资本500万元以上私营企业800家。

进一步优化公共服务。巩固各类社会保险全覆盖成果，全面实施医疗、生育保险市级统筹，提高大病医疗保险报销标准和新农合参合人员受益水平。以提高质量为核心，以促进公平为重点，科学配置教育资源，加强师德师风建设，努力办好人民满意的教育。深入实施基本药物制度，探索推进市区医疗卫生资源整合，继续实施社区卫生服务机构提升工程，加快建设覆盖城乡的公共卫生服务体系。统筹城乡客运发展，全市行政村客运班车“村村通”，实现市区城乡公交一体化，力争市区公交出行分担率提高3个百分点。健全住房保障体系，全市新增保障性住房10225套（间），市区新开工建设拆迁安置房255.5万平方米。扩大住房公积金制度惠及面，新增开户缴存职工3万人。大力发展社会养老服务，出台扶持民办养老机构政策措施，筹建老年社区，新建社区居家养老服务中心（站）260家。

认真实施民生工程。加大民生投入，多办实事好事，切实维护群众切身利益。扩大企业工资集体协商制度覆盖面，不断提高职工工资收入水平。大力推行“阳光拆迁”，全面实施房屋征收补偿安置办法，调整完善服务管理体系，认真执行新被征地农民社会保障“即征即保”制度。落实贫困家庭子女助学和孤儿教育、医疗、就业等各项保障政策，做好有劳动能力残疾人就业安置工作。推进社会福利适度普惠，继续发展慈善公益事业，加快泰州福利院、高港养护院建设。加强法律援助工作，进一步扩大覆盖范围。抓好“米袋子”、“菜篮子”，稳定物价总体水平，落实社会救助和社会保障标准与物价上涨挂钩联动机制，确保困难群众生活不因物价上涨而受影响。继续办好农村新一轮实事，启动实施经济薄弱地区和贫困农户脱贫奔小康工程，推进农村饮用水安全工程建设，再解决14.6万人安全饮水问题。

2. 调整优化结构，推动产业转型。把产业结构战略性调整作为经济转型升级的重中之重，不断加快新型工业化进程，着力提升产业发展层次和整体竞争力。

加快建设产城一体的医药名城。继续举全市之力建设中国医药城，以医药高新区的发展引领经济转型升级。年内力争完成固定资产投资150亿元，落户企业250家，其中世界前20强医药企业4-5家，引进各类高层次人才600名，当好全市转型升级的先导示范区。突出项目招引和企业集聚，推进一批在谈跨国医药企业落户，促进一批重大产业项目建成投产，推出一批具有自主知识产权、填补国际市场空白的重大新产品投产，培植20家年产值超10亿元的“小巨人”企业。突出多元融资和资本集中，积极创建科技金融示范区，完善融资平台，推进知识产权质押，壮大担保公司规模，加强与全球知名基金公司合作，力争形成百亿级产业基金规模，继续推动企业上市，建立金融创新活跃、服务功能齐全、支持重点突出的现代资本体系。突出功能完善和载体集成，加快厂房集聚区、康健医疗区、原料药基地、数据产业园二期等产业载体建设，加快疫苗工程中心、分子诊断平台、医疗器械平台、动物实验中心等公共服务载体建设，加快城市综合体项目以及综合服务楼、康居社区一期等城市载体建设，着力打造富有现代气息的产业新城。

积极发展新兴产业和优势特色产业。深入实施“新兴产业双倍增计划”，加快形成市场规模优势和技术领先优势。围绕生物医药产业化，招引实施一批高端医药生产型项目、知名药企总部型项目。围绕纬创等龙头企业，招引实施一批配套产品和半导体封装、新型元器件等项目。围绕促进新能源产业链发展，招引实施一批光伏应用产品、储能电池设备、新能源技术研发等项目。培育壮大新材料、环保装备等国内领先的新兴产品集群，形成一批新兴产业后备梯队。积极运用高新技术和先进适用技术改造提升传统产业，推进机电、船舶、化工、不锈钢等传统产业向产业链高端攀升，注重引导船舶企业向节能环保船舶和高附加值的海洋工程、特种船舶领域拓展。围绕产品高端化、装备高新化及优势产能扩张，加大有效投入，全年实施亿元以上工业技改项目220个。扎实开展“质量强市”工作，认真评审“市长质量奖”。深入推进企业信息化系统建设，组织实施核心港区和城北物流园区的“两化融合”省级示范（试验）区工程，力争新增省示范（试点）企业20家。进一步加快建筑业发展，推动建筑企业经营管理和施工方式创新，建筑业总产值增长12%。

提速发展现代服务业。继续抓好“833工程”，加快实施供销产业园、天星洲物流开发、山煤集团煤炭储备、雨润冷链仓储物流等重大项目，力争新增营业收入超100亿元的集聚区1-2家、超10亿元的企业2-3家。着力构建“四大板块”，加快大型商贸中心和新兴业态发展，打造中心城市核心商务板块；大力发展医药研发、会展交易、数据服务、服务外包等知识技术密集型产业，打造医药高新区高端服务板块；做大做强泰州港综合物流园、苏中沿江化工物流园、江苏三江现代物流中心，打造沿江现代港口物流板块；实施凤城河、泰州华侨城、溱湖、千岛菜花和李中水上森林、秋雪湖等景点景区的深度开发，加强重大旅游项目的招引、合作，打造“水城慢生活”生态休闲旅游板块。推进工贸企业分离，加快发展生产性服务业。积极发展现代金融服务业，力争新引进全国性股份制商业银行1-2家。

大力发展现代农业。深入实施“5218提升工程”，推动农业结构调整，加快形成农业现代化体系。提高农业综合生产能力，扎实开展高产增效创建，建设50个部省级万亩粮油高产创建示范片。加快农业产业化进程，打造一批现代农业产业园区、农产品加工集中区和区域性农产品交易市场，争创1个国家级现代农业示范区。推进农业品牌化建设，实现主要农产品生产标准全覆盖，全市认定“三品”基地占耕地面积达80%以上，争创1-2个省级以上农业标准化示范区。提高农业装备化水平，加快高标准农田和农业机械化建设，实施好机电泵站改造、高效节水灌溉等农田水利工程。强化农业技术集成与创新示范，实施“农业重大技术推广计划”。坚持农业集约化发展，加强耕地特别是基本农田保护，推进万顷良田建设，新增适度规模经营面积16万亩、高效设施农业面积8万亩、农村三大合作组织250家。

3. 深化开放创新，增强发展动力。把开放创新作为推进经济转型升级的主动力，充分利用“两个市场、两种资源”，加快集聚创新资源、高端要素，不断优化体制机制、运行体系，努力在更广领域、更高层次上参与竞争与合作。

强力推进招商引资。进一步加大行政推动力度，切实提高招商活动实效。紧紧围绕新兴产业和优势特色产业，大力招引重大投资项目和拥有自主知识产权的项目，广泛吸纳有助于提升产业竞争力的优质资源要素。着力转变招商方式，建立高层次招商信息网络，更加突出产业链招商和港台、日韩、欧美等重点区域招商。积极引导重点企业深化与跨国公司、央企的战略合作，通过并购重组实现高位嫁接。办好第六届中国生物产业大会、第三届中国（泰州）国际医药博览会等重大活动，争取在重大产业项目招引上取得新突破。全年力争引进超3000万美元项目25个，其中过亿美元项目5个。

奋力扩大外贸出口。进一步优化出口结构，提高一般贸易、服务贸易、自主知识产权和自主品牌产品出口比重，力争机电和高新技术产品占外贸出口的70%以上，年出口超3000万美元的企业20家以上。引导出口企业强化品牌建设，鼓励有条件的企业申请商标国际注册、参与各类标准的制订修订。加快企业“走出去”步伐，支持重点企业开展境外资本运营，开辟国际化经营新空间。推进核心港区建设，不断完善基础设施，提高港口吞吐能力。加强外事、侨务工作，促进对外交流合作。

构建区域创新体系。实施“十百千创新型企业培育计划”，促进高新技术企业快速成长，新增市级以上创新型企业20家、高新技术企业50家、高新技术产品150个。进一步完善“一中心五平台”服务功能，鼓励企业组建产业技术创新联盟，推广建立国家和省级企业技术中心，推动大企业与知名院校共建工程（技术）研究中心，建立多层次的科技孵化平台。充分发挥国家专利战略推进与服务中心作用，鼓励企业通过购买、重组、特许经营等方式引进优质知识产权。深入实施“凤城千人计划”，全力打造中国医药城“人才特区”，精心组织“百名海外博士泰州洽谈会”等活动，力争全年引进高层次人才1200名，其中海外优秀人才250名。

转变园区发展方式。引导各地抓紧编制高起点发展规划、大力招引高质量产业项目、积极构建高层次发展平台，着力在全市打造一批特色产业园区，促进经济转型升级。加快推进泰州经济开发区向以光电产业为主导的国家级出口加工区和保税园区转型，靖江经济开发区向以高端装备制造业为主导的国家级开发区转型，泰兴经济开发区向循环经济园区转型，姜堰经济开发区向智能电网产业园区转型，兴化经济开发区向食品加工产业园区转型，海陵工业园区向新能源产业园区、文化创意产业园区转型，高港高新区向以通用动力、墙纸为重点的特色产业园区转型，市农业开发区向国家级现代农业科技示范园区转型，城北物流园区向以现代物流为主导的多式联运枢纽转型。

深化重点领域改革。构建更加灵活高效的市区城建管理体制，进一步促进海陵区、医药高新区、高港区三区联动融合。稳定农村基本经营制度，扩大农村土地承包经营权确权登记试点。积极实施农村集体土地流转，探索承包地、宅基地空间置换等多种形式。继续开展市区村组（社区）股份制改革，抓好街道社区调整建设工作。深化医药卫生体制改革，完善人事分配制度，继续推进公立医院改革试点。巩固事业单位机构设置和人员编制清理规范成果，实施事业单位分类改革和人事制度改革。积极构建财政资金绩效管理机制和评价体系，对市级财政专项资金项目全部实施绩效目标管理。

4. 统筹协调发展，建设美好城乡。以新型城镇化为导向，以生态名城建设和美好城乡建设行动为抓手，更高层次统筹区域发展，大力推进城乡发展转型。

加快中心城市建设与发展。着力推进中心城市向建管并重、更加注重内涵和功能提升转变。进一步完善路网结构，加快推进231省道海陵至高港段、海军大道和迎春东路东延、梅兰路接通、东风路南延、鼓楼路北延等道路建设。进一步提升城市功能，建成长江大桥和机场，全面建成文化中心，加快实施金融服务区、华润万象城、茂业天地、泰茂城、明发、台湾国际新城以及人民医院新院、新区高级中学等重点工程，开工建设体育公园，高标准建设垃圾焚烧发电厂，继续实施城市电网、老小区管道燃气改造和城区污水管网建设，抓紧市区供水“浑改清”和三水厂改扩建前期工作，加强人民防空体系和防空防灾一体化建设。进一步发展城市水利，继续推进城市防洪和水生态环境工程建设，启动城区西北片河道及稻河、草河、老通扬运河治理，加快实施卤汀河、泰东河、引江河拓浚工程。以数字化、网格化管理为抓手，进一步提高城市管理精细化水平。

加快城乡一体化步伐。加强和改进对县域经济的支持和指导，推动区域差异化、特色化发展。推进城乡规划、产业布局、基础设施、公共服务、劳动就业“五个一体化”，深化乡镇综合改革试验示范区建设。深入实施“125工程”，加快戴南、溱潼、黄桥、新桥等十个小城市（城市片区）建设步伐，鼓励其他有条件的城镇向小城市方向发展。完善户籍制度，放宽城镇落户条件，引导农民逐步有序融入城镇；推行居住证制度，积极实施新市民工程。抓好全面小康村建设“十百千提升工程”，创建全面小康达标村、示范村各100个以上。强化农村环境综合整治，完成20%村庄整治任务，健全农村卫生保洁长效机制和生活垃圾、生活污水集中收集处理体系。

加快建设生态名城。深化国家生态市创建工作，确保通过国家环保模范城市复核验收，姜堰、海陵达到国家生态市（区）创建标准，全市50%的乡镇力争进入国家生态乡镇行列。注重生态景观打造，扎实抓好中心城市北部生态区、国家级生态工业示范区、原生态长江绿带和大桥生态景观公园等建设。加大环境保护力度，有效控制农业面源污染，大力开展绿色通道、村庄绿化、农田林网建设，积极推动各类工业园区生态化建设和改造，强化大气污染综合防治，继续加强秸秆禁烧和综合利用。着力推进可持续发展，强化节能减排，淘汰落后产能，在用能企业大户中积极开展能源管理体系认证，加快发展循环经济、低碳经济和节能环保产业，进一步加大公共机构节能技改力度。

5. 推进文明进步，保持和谐稳定。突出文化发展转型和社会管理创新，着力营造特色鲜明的文化环境，积极塑造奋发向上的精神风貌，继续保持安定团结的良好局面。

加快建设文化名城。出台实施“文化名城建设行动计划”，加快繁荣文化事业、发展文化产业、培育文化人才、彰显文化特色。坚持城市建设与文化建设相融合，科学保护文化遗产，着力传承历史文脉，启动建设评书评话、盐税等特色博物馆，加快推进五巷涵西、城中、柴墟、海军舰艇主题文化公园，以及黄桥、溱潼古镇和兴化金东门等重点街区景区建设，打造1-2个中国历史文化名街和一批富有特色的文化交易市场。坚持社会效益与经济效益相统一，把文化产业作为优先发展产业，落实扶持政策，着力培育文化创意产业园等特色文化产业园区；扶持成长型文化产业发展，力争文化产业规模两年倍增。坚持文化事业与文化惠民相促进，完善公共文化服务体系，全面建成博物馆、图书馆、美术馆、文化馆新馆和青少年活动中心，实现各类公共文化设施免费向市民开放，推动兴化市创建省级公共文化服务体系建设示范区；扶持文艺创作，推出更多群众喜闻乐见的优秀作品。深化文化体制改革，健全文化人才选用和激励机制，营造有利于出精品、出人才、出效益的良好环境。办好水城水乡国际旅游节、梅兰芳艺术节等重要活动，承办好省第七届农民运动会，办好市第四届运动会。

提升现代文明素质。加强社会主义核心价值体系建设，大力弘扬“三创三先”精神。进一步加强道德规范约束，深入开展社会公德、职业道德、家庭美德和个人品德教育。加强未成年人思想道德建设，重点关心帮助留守儿童和贫困家庭、单亲家庭、外来务工家庭子女，让每一位青少年都能健康成长。加快建设社会信用体系，强化重点领域和重点行业的信用管理，营造良好的诚信秩序。深化文明城市创建，放大系列品牌效应，广泛开展群众性精神文明创建活动，使全市人民精神风貌更加昂扬向上。积极开展双拥共建活动，实施退役士兵城乡一体化安置，做好国防教育、国防动员、民兵预备役和兵员征集工作。

加强和创新社会管理。积极打造社会管理示范区。推广镇村户三级联动服务管理体系和治安中心户长制等有效机制，落实网格化服务管理模式，积极推动社会服务管理中心、社会志愿者和社工队伍建设；充分发挥社区在服务居民、维护稳定等方面的社会事务平台作用，落实好驻社区单位的共建责任，切实解决城中村、城郊结合部管理难问题。积极打造和谐稳定示范区。落实社会稳定风险评估制度，推进现代化治安防控体系建设，突出抓好企业生产安全、社会公共场所安全、食品药品安全、校车校舍校园安全，建成“全省平安市”；落实信访维稳责任制，坚持“八访”工作法，深化大调解机制建设，努力预防和化解各类矛盾纠纷。积极打造法治建设示范区。加强“法治城市”建设，全面启动争创全国“六五”普法先进市活动，抓好社区矫正和刑释解教人员安置帮教工作，重点实施法治惠民十大工程，进一步营造公平正义的法治环境；坚持“四民主、两公开”，扎实开展村民直评村官暨勤廉指数测评、基层直评机关等活动，深化民主法治示范村（社区）创建，不断推进基层民主法制建设。

6. 强化自身建设，提高行政效能。争创全省转型升级示范区，政府工作首先要转型升级。我们将扎实推进服务型政府建设，大力改进各级机关干部的思想作风和工作方法，努力以能力素质、行政效能的大提升为转型升级提供坚强保证。

创新政府服务，增强发展推动力。加强学习型机关、学习型班子建设，提高驾驭复杂局面、承担繁重任务、推动科学发展的能力和水平。继续深化“三服务”活动，帮助企业和投资者化解土地、资金、用工等要素制约。深化政银企合作，引导金融机构主动顺应货币政策调整方向，推动金融创新发展，促进更多信贷资金投向实体经济。充分发挥政策的引领和杠杆作用，加强对各类优惠政策的归并、集成；探索设立政府创投引导资金，扶持设立创业投资企业，吸引民间资本跟进投入，引导社会资本投向新兴产业和高新技术企业。优选项目，积极稳妥地做好企业债券相关工作。完善行政审批机制，扎实抓好“三集中三到位”、并联审批、网上审批、招投标、限时办结等各项工作，千方百计提高行政效能、降低行政成本。

加强效能建设，增强行政执行力。坚持依法行政，严格按照法定权限和程序行使权力、履行职责，切实加强对行政权力的监督制约。认真对照本届政府任期目标，狠抓工作推进和落实，确保完成各项目标任务。完善政府常务会议定期汇报和研究分析制度，加大对重点工作、重大工程和重要民生项目的跟踪督查力度。推进统计“四大工程”建设，做好基本现代化进程监测和省定“八项工程”相关监测统计。深化拓展“三解三促”活动，着力提升“12345”政府服务热线、市长信箱、“行风热线”办理质量，真心实意、尽心尽力解决群众反映的矛盾和问题。强化效能监察，加大行政问责力度，坚决把各项决策部署落到实处。

推进科学施政，增强政府公信力。自觉接受市人大及其常委会的法律监督、工作监督和市政协的民主监督，主动听取各民主党派、工商联、无党派人士以及工会、共青团、妇联等人民团体的意见和建议。加强与人大代表和政协委员的联系，认真办理人大代表建议和政协提案。坚持问政于民、问需于民、问计于民，深入开展调查研究，大力推进网络问政，加快建立更加科学的决策机制，使政府的工作部署、政策措施更加符合客观实际和群众意愿。深化政务公开，加强政府新闻发布工作，全面提升行政权力网上公开透明运行效能。严格控制“三公”经费，实行一般性行政经费零增长，加强对政府性资金、投资项目的审计监督和领导干部的经济责任审计。严格执行党风廉政建设责任制，不断完善惩治和预防腐败体系。

各位代表，新的一年，我们面临着新的挑战和考验，肩负着重大的责任和使命。让我们紧密团结在以胡锦涛同志为总书记的党中央周围，高举中国特色社会主义伟大旗帜，坚持以邓小平理论和“三个代表”重要思想为指导，深入贯彻落实科学发展观，在中共泰州市委的坚强领导下，坚定信心，求真务实，攻坚克难，奋力争创全省转型升级示范区，努力走好具有时代特征、泰州特色的现代化建设之路，以优异成绩迎接党的十八大胜利召开！
